# Removal of Nickel from Aqueous Solutions by Natural Bentonites from Slovakia

**DOI:** 10.3390/ma14020282

**Published:** 2021-01-07

**Authors:** Matej Šuránek, Zuzana Melichová, Valéria Kureková, Ljiljana Kljajević, Snežana Nenadović

**Affiliations:** 1 Department of Chemistry, Faculty of Natural Sciences, Matej Bel University, Tajovskeho 40, 97401 Banská Bystrica, Slovakia; matej.suranek@student.umb.sk; 2 Department of Hydrosilicates, Institute of Inorganic Chemistry, Slovak Academy of Sciences, Dubravska cesta 9, 84536 Bratislava, Slovakia; valeria.bizovska@savba.sk; 3 Department of Materials, Vinča Institute of Nuclear Sciences-National Institute of the Republic of Serbia, University of Belgrade, 11000 Belgrade, Serbia; ljiljana@vin.bg.ac.rs (L.K.); msneza@vin.bg.ac.rs (S.N.)

**Keywords:** nickel, bentonite, adsorption, Langmuir isotherm, thermodynamic parameters

## Abstract

In this study, the removal of nickel (Ni(II)) by adsorption from synthetically prepared solutions using natural bentonites (Lieskovec (L), Hliník nad Hronom (S), Jelšový Potok (JP), and Stará Kremnička (SK)) was investigated. All experiments were carried out under batch processing conditions, with the concentration of Ni(II), temperature, and time as the variables. The adsorption process was fast, approaching equilibrium within 30 min. The Langmuir maximum adsorption capacities of the four bentonite samples used were found to be 8.41, 12.24, 21.79, and 21.93 mg g^–1^, respectively. The results best fitted the pseudo-second-order kinetic model, with constant rates in a range of 0.0948–0.3153 g mg^–1^ min. The effect of temperature was investigated at temperatures of 20, 30, and 40 °C. Thermodynamic parameters, including standard enthalpy (Δ*H^0^*), Gibbs energy (Δ*G^0^*), and standard entropy (Δ*S^0^*), were calculated. The adsorption of Ni(II) by bentonite samples was an endothermic and spontaneous process. These results indicated that, of the bentonite samples used, the natural bentonites from JP and SK were most suitable for the removal of nickel from synthetically prepared solutions.

## 1. Introduction

The contamination of the environment with wastewater has received a great deal of attention in recent years. Wastewater contamination consists of fouling by heavy metal ions, which originate from various industrial activities; however, sometimes an increased concentration of heavy metals may also be found in drinking water sources. Heavy metals pose an extraordinary environmental risk, due to their ability to accumulate in living and nonliving organisms [1,2].

The group of heavy metals posing a potential risk to the environment also includes nickel. Nickel (Ni, atomic number: 28) is a silver-white heavy metal, occurring in oxidation states of −1, 0, +1, +2, +3, and +4. The most common and important oxidation state of Ni is +2. In nature, this occurs naturally in soils and surface waters (at less than 100 mg L^–1^) [3]. The source of nickel in wastewater is most often as waste from electroplating and battery production. Its other sources include metal mining, smelting, fossil fuel combustion, vehicle emissions, domestic, municipal, and industrial waste disposal, fertilizer applications, and organic fertilizers [4]. Although nickel (as Ni(II) complexes) is an essential element for plants and plays an important role in plant metabolism at increased concentrations (5–15 mg L^–1^ Ni, over one week), it causes chlorosis, necrosis, and leaf wilting [3]. As nickel is a ubiquitous metal, contact with nickel compounds (soluble or insoluble) cannot be eliminated. The most common skin allergies are dermatitis, while diseases such as cardiovascular and kidney diseases, lung fibrosis, and lung and upper respiratory cancer are also known. It is now confirmed that some nickel compounds are highly carcinogenic to humans, but the mechanism of carcinogenesis is unclear [5].

Ni(II) ions are also very important parts of rechargeable alkaline Zn–Co batteries. The electrochemical performance of the battery can be much higher via substitution of Ni(II) ions onto a Co_3_O_4_ electrode. The substitution ratios of Ni(II) ions can be different. The study [6] writes about the methods of doping the Co_3_O_4_ electrode with nickel ions. Cheap and effective electrocatalysis is crucial for hydrogen production via electrocatalytic water splitting. Nickel is used as a part of Ni/Gd_2_O_3_/NiO nanofibers, which have very high electrocatalytic performance. The core of this material is built by NiO, and the molecules of Ni/Gd_2_O_3_ are concentrated around the core. The details are summarized in the study [7].

As the concentration of nickel is constantly growing, it is important to know and explore all of its sources, so that technologies can be developed to reduce its concentration in the environment.

With the increasing concentration of toxic metals in wastewater and the stringent standards that determine the maximum concentration of heavy metals in wastewater, a great emphasis is placed on research into materials suitable for the cheap and efficient removal of heavy metals from wastewater. Several methods are known for removing toxic metals from water, such as ion exchange [8], chemical precipitation [4], membrane filtration [9], reverse osmosis [10], solvent extraction [11], electrochemical treatment [12], and adsorption [13]. The removal of metal ions by adsorption is of wide research interest, as it is a relatively cheap and easy-to-implement method [14]. Different materials may be used as adsorbents for metal ions and may be obtained from organic, biological, or mineral sources (e.g., agricultural waste, aquatic and terrestrial biomass, biochar [15] naturally occurring in soil and mineral deposits, and other locally available waste materials) [16].

Natural clays (particularly bentonite) have received significant attention for heavy metals adsorption from contaminated water due to their applicability. Bentonite has several advantageous properties as an adsorbent, including low cost, good selectivity, and ion exchange capacity and regenerability [17,18]. Bentonite is used for adsorption of Li [19], Cs [20], Fe(III) [21], Cd(II), Zn(II) [22], Cu(II), Pb(II), and Ni(II) [23]. The adsorption of Ni(II) was studied on Slovak bentonites [24,25], Iranian bentonites [26], nano-bentonites [27], fungal dead biomass composites with bentonite [28], hybridized bentonites [29], and other materials.

This research is devoted to the study of nickel adsorption on bentonites from selected Slovak deposits with different structures and chemical compositions, in order to compare their adsorption efficiencies as a function of different nickel concentrations. The influences of the temperature, time, and initial concentration of Ni(II) on the course of sorption were monitored, and for better illustration of Ni(II) adsorption on natural bentonites, thermodynamic parameters were also calculated.

## 2. Materials and Methods

### 2.1. Adsorbent Properties and Preparation

Four different bentonite samples were examined for the removal of Ni(II) from synthetically prepared aqueous solutions. All bentonite samples came from Central Slovakia, specifically from the Lieskovec deposit (L), the Jelšový Potok deposit (JP), the Stará Kremnička deposit (SK) (near the Jelšový Potok deposit), and the Hliník nad Hronom deposit (S).

All bentonite samples were used without further purification. Bentonite samples were sieved using a standard mesh sieve (<200 μm) and dried at 105 °C for approximately 2–3 h in Petri dishes in a drying oven. Then, the samples were transferred into polypropylene bags and stored in a desiccator until further use. The samples were tested for Ni(II) adsorption, without any pretreatment.

### 2.2. Synthetic Solutions

All chemicals used were of analytical reagent grade and were used without further purification. The aqueous solutions of nickel (NiSO_4_·7H_2_O, for analysis (CAS No. 10101-98-1) Acros Organics, NJ, USA) were prepared with water deionized via reverse osmosis (Demiwa, Watek, Czech Republic).

A certified reference material—aqueous Ni solution with a concentration of 1.0 g L^–1^ (matrix 2% HNO_3;_ Slovak Metrology Institute, Bratislava, Slovakia)—was used for preparing standard metal ion solutions, which were used for AAS analysis.

### 2.3. Adsorption Experiments

The adsorption experiments were carried out in 250 mL Erlenmeyer flasks, by mixing 0.25 g of an adsorbent (weighed to four decimal places) with 50 mL of a nickel solution. The contents of the Erlenmeyer flasks were then shaken at a given temperature (20, 30, and 40 °C) in an ES–20/60 (bioSan, Riga, Latvia) orbital shaker at 200 rpm. The suspensions were centrifuged after 2 h or after the desired contact time in the case of time dependence experiments. Each adsorption experiment was performed in duplicate using two independent samples. Atomic absorption spectrometry was used for the determination of nickel content in the solutions. The limit of experimental error between duplicates was less than ±5%.

The adsorption percentage (Ads.%) was calculated by Equation (1):

(1) $$\mathrm{Ads}.\;\%\;=\;\frac{c_{o}\;-\;c_{t}}{c_{o}}\;\times \;100$$

where *c_o_* is the initial Ni(II) concentration (mg L^–1^) and *c_t_* is the Ni(II) concentration left in aqueous solutions at time *t* (mg L^–1^).

The amount of the adsorbed Ni(II) per mass unit of the adsorbent at time *q_t_* (mg g^–1^) was calculated according to Equation (2):

(2) $$q_{t}\;=\;\frac{\left(c_{o}\;\;{-\;c}_{t}\right)V}{m}$$

where *V* is the volume of the aqueous phase (L) and *m* is the amount of the bentonite (g).

### 2.4. AAS Analysis

The Ni(II) concentrations before and after adsorption were measured using an atomic adsorption spectrometer AVANTA Σ (GBC Scientific, Melbourne, Australia) with acetylene–air flame atomization. The data were processed using GBC Avanta v.2.0 software. The working wavelengths were 341.5 nm for a Ni(II) concentration up to 20 mg L^–1^ and 351.5 nm for a Ni(II) concentration up to 300 mg L^–1^. Standard metal ion solutions were used for periodically checking the instrument response.

### 2.5. FTIR Analysis

For a more detailed qualitative analysis of the adsorption materials used, their IR spectra were measured at the Department of Hydrosilicates IIC SAS in Bratislava, Slovakia. The spectra in the mid-IR region (MIR, 4000–400 cm^–1^) were obtained on a NICOLET 6700 FT-IR spectrometer (Thermo Scientific, Waltham, MA, USA) equipped with a KBr beam splitter and a DTGS detector. Samples were measured by the KBr pressed disk technique, where 1 mg sample was homogenized with 200 mg KBr. The pellets were heated overnight at 130 °C in order to minimize the content of adsorbed water. Each spectrum was the average of 128 scans with a resolution of 4 cm^–1^. The Thermo Scientific OMNIC™ v.7.2 software package was used for the measurement and evaluation of the IR spectra.

## 3. Results and discussion

### 3.1. Characterization of the Adsorbents

#### 3.1.1. Chemical Analysis and X-Ray Analysis

The chemical compositions of the used bentonite samples have been described in detail in previous works [24,25,30,31,32] and are shown in Table 1. The JP sample contained added Na_2_CO_3_, unlike other bentonite samples used. It was shown previously by X-ray analysis (Table 2) that montmorillonites (JP 86% [24], L 64% [25], S 47% [30], and SK 85% [30]) were the dominant component in all the samples. The samples also contained quartz, kaolinite, and biotite in smaller amounts. Opal-C (20%) was found in sample S [31].

#### 3.1.2. IR Spectroscopy

**Figure 1:**
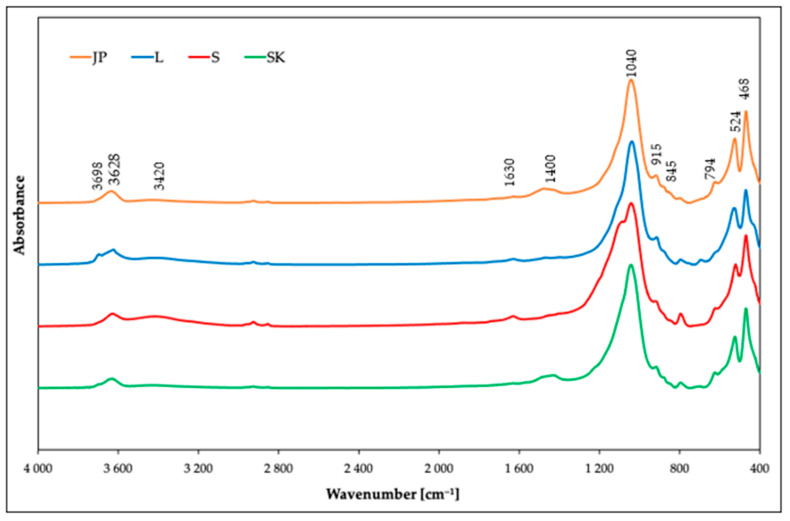
IR spectra of the used bentonites before the adsorption of Ni(II) ions.

FTIR spectroscopy in the MIR region was used for the structural characterization of the studied samples. The IR spectra (Figure 1) revealed the dominant component was smectite in all the samples. The presence of the smectite mineral montmorillonite was proved by the peak at 3628 cm^–1^, which corresponded to the stretching vibrations of the structural OH groups of montmorillonites. The bending vibration modes of the structural OH groups were observed at 915 cm^–1^ (AlAlOH), 845 cm^–1^ (AlMgOH), and 882 cm^–1^ (AlFeOH). The substitution of Fe by Al was observed mainly in the JP and SK samples. In the S and L samples, the intensity of the bending vibration mode of AlFeOH was negligible. The complex band near 1040 cm^–1^ and the shoulder near 1100 cm^–1^ corresponded to the stretching vibrations of Si–O groups. The bending modes of Al–O–Si and Si–O–Si were observed at 524 and 468 cm^–1^, respectively [33]. The bands near 3420 and 1630 cm^–1^ appeared due to the presence of molecular water [34]. In addition to the vibration bands of montmorillonites, the IR spectroscopy also revealed the presence of admixtures. The shoulder at 3698 cm^–1^ corresponded to the stretching vibration of kaolinite, observed mainly for sample L, although a weak shoulder was also observed in sample SK. The presence of another admixture proved the band near 794 cm^–1^ originated from the stretching vibrations of Si–O groups, from either quartz or silica. This band was observed in all the samples, but most intensively in sample S. The broad band near 1400 cm^–1^ in the IR spectra of the JP and SK samples also indicated the presence of carbonate [35,36,37].

The IR spectra of samples after the adsorption were also measured (Figure S1). Differences in the spectra before and after sorption were only negligible, due to the unchanged structure of montmorillonites after the sorption of Ni(II). It can be observed only in the absence of vibration around 1400 cm^–1^.

### 3.2. Effect of Metal Concentrations

The effects of the initial Ni(II) concentration on the adsorption capacities of the used bentonite samples were investigated by varying the initial concentration of Ni(II) from 50 to 300 mg L^–1^ in solutions, at temperatures of 20, 30, and 40 °C. The amount of the samples (0.25 g per 50 mL of the solution) was constant, and the pH was not adjusted from its value of 5.85.

**Figure 2:**
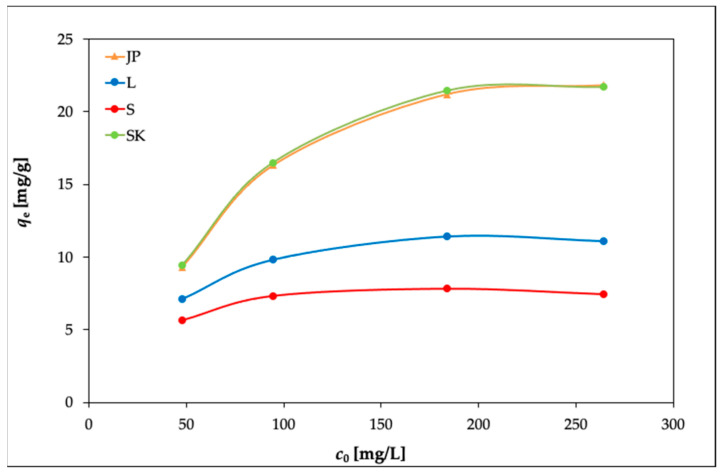
Effects of the initial concentration of Ni(II) on the adsorption capacities of the bentonite samples (experimental conditions: 50 mL solution; 0.25 g bentonite; temperature, 20 °C).

**Figure 3:**
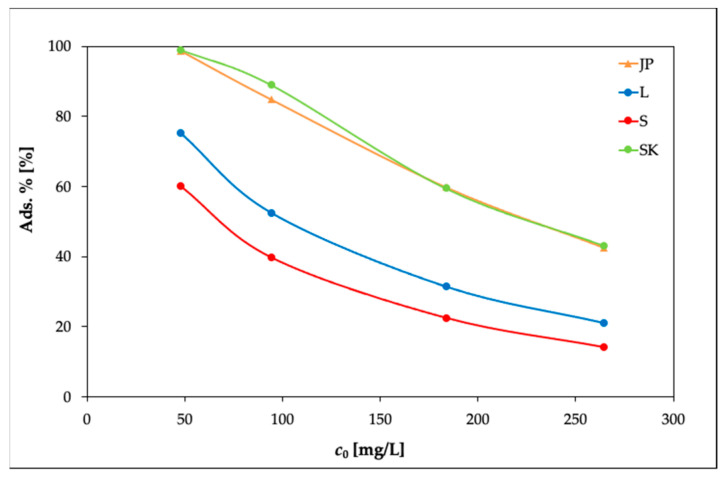
Effects of the initial concentration of Ni(II) on the adsorption percentage of the bentonite samples (experimental conditions: 50 mL solution; 0.25 g bentonite; temperature, 20 °C).

**Figure 4:**
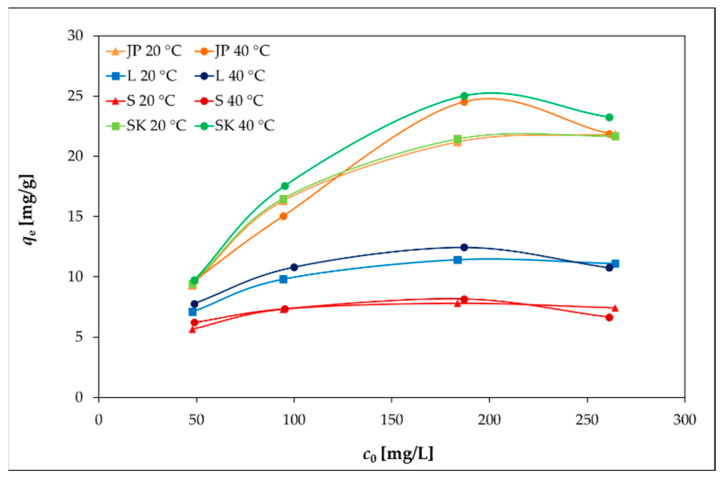
Effects of the temperature and the initial concentration of Ni(II) on the adsorption capacities of bentonite samples.

The results are presented in Figure 2, Figure 3 and Figure 4. In Figure 2, it can be seen that the equilibrium adsorption capacities of the bentonite samples toward Ni(II) increased while the adsorption percentages of Ni(II) (Ads. %) in Figure 3 showed an opposite trend.

However, at 40 °C (Figure 4), the removal efficiency slightly decreased with the increasing initial Ni(II) concentration, as the concentration gradient increased, leading to a higher probability of collision between Ni(II) and active adsorption sites on the bentonite and thereby increasing the adsorption capacity. With a further increase in the concentration of Ni(II), the active adsorption sites became saturated [38,39].

The elevated temperature caused only a slight increase in *q_e_* for all four bentonites, as can be seen in Figure 4.

### 3.3. Adsorption Isotherms

The adsorption data were described using the Freundlich and Langmuir adsorption models, which were applied in a linear form, according to Equations (3) and (4):

(3) $$\mathrm{Freundlich}\;\mathrm{isotherm}:\;{\mathrm{logq}}_{e}{=\;\mathrm{logK}}_{F}\;+\;\frac{1}{n}{\;\mathrm{logc}}_{e}$$

(4) $$\mathrm{Langmuir}\;\mathrm{isotherm}:\;\frac{c_{e}}{q_{e}}\;=\;\frac{1}{b\;q_{m}}\;+\;\frac{1}{q_{m}}c_{e}$$

where *q_e_* is the amount of Ni(II) adsorbed per unit weight of bentonite at an equilibrium concentration (mg g^–1^), *q_m_* is the maximum adsorption capacity corresponding to the monolayer adsorption capacity (mg g^–1^), *c_e_* is the equilibrium concentration of Ni(II) in the solution (mg L^–1^), *1*/*n* is an empirical parameter related to the intensity of adsorption (where for values in the range of 0.1 < *1/n* < 1, adsorption is favorable), *K_F_* is the surface adsorption equilibrium constant (mg g^–1^) and *b* is the Langmuir coefficient which represents the equilibrium constant related to the adsorbate–adsorbent affinity (L mg^–1^).

**Figure 5:**
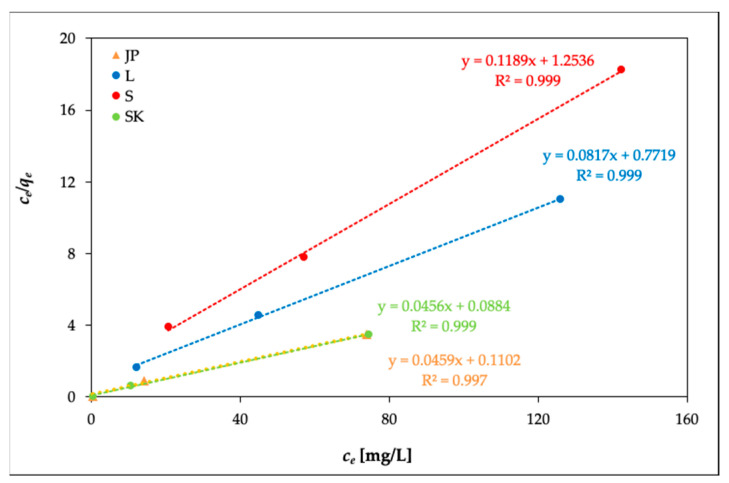
Model of Langmuir isotherms (temperature: 20 °C).

The experimental data were plotted for Freundlich isotherms as log *q_e_* vs. log *c_e_* and for Langmuir isotherms as *c_e_/q_e_* vs. *c_e_*. The adsorption constants *K_f_* and *n* (for Freundlich isotherms) and *q_m_* and *b* (for Langmuir isotherms) were calculated from these linear plots. The evaluation of isothermal equations was performed on the basis of correlation coefficient *R^2^*. Slightly improved results for the Ni(II) adsorption on the studied samples were provided by the Langmuir model (Figure 5) (*R^2^* = 0.988–0.999) vs. the Freundlich model (*R^2^* = 0.922–0.999). Table 3 shows the calculated results and *R^2^*. The values of *q*_m_ were dependent on temperature; with the increasing temperature, *q*_m_ slightly increased.

The dimensionless separation factor *R_L_* defines the nature of adsorption in the Langmuir isotherm and can be calculated by Equation (5) [40]:

(5) $$R_{L}\;=\;\frac{1}{1\;+\;b\cdot {\;c}_{0}}$$

where *b* is the Langmuir equilibrium constant (L mg^–1^) and *c_o_* is the initial concentration of Ni(II) (mg L^–1^). *R_L_* values reveal whether the adsorption is favorable (0 < *R_L_* < 1), unfavorable (*R_L_* > 1), linear (*R_L_* = 1), or irreversible (*R_L_* = 0) [41].

The *R_L_* values at concentrations of 50–300 mg L^–1^ Ni(II) for the used bentonites (Table 4) indicate that the Langmuir model is favorable as indicated by 0 < *R_L_* < 1; however, the Freundlich model also implies favorability as indicated by 0.1 < 1*/n* < 1.

The comparisons of the *R^2^* values for both models showed that there were no significant differences between the Langmuir model and the Freundlich model. The Freundlich model is valid for heterogeneous surfaces, while the Langmuir model is preferred for monolayer adsorption onto a surface, on which the binding sites have an equivalent affinity for adsorption, with a finite number of identical sites [41].

The agreement of the experimental data with the Langmuir isotherm indicated a homogeneous adsorption process, which leads to monolayer binding.

Table 5 compares the maximum adsorption capacities of different natural and modified bentonites for Ni(II) removal reported in previous studies. 

Among the natural materials, nano-bentonite showed the highest maximum adsorption capacity. The measured adsorption capacities of bentonites in this work towards Ni(II) are comparable to [43] or higher than the values reported by others [44].

### 3.4. Kinetic Studies

**Figure 6:**
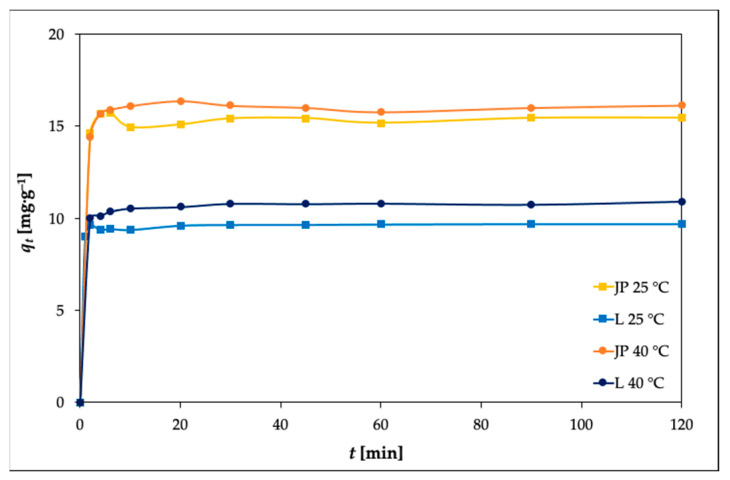
Effects of adsorption time at 25 and 40 °C on the adsorption of Ni(II) by the JP and L bentonites (experimental conditions: 50 mL solution; 0.25 g adsorbent; *c_0_* 100 mg L^–1^; pH 5.85).

The effects of the contact time on the adsorption of Ni(II) by the JP and L bentonites were investigated at various time intervals (2–120 min), and the results are shown in Figure 6.

The adsorption rate was found to be rapid during the first 10 min of adsorption. This is in agreement with previous studies [23,45], where fast adsorption of metal ions has been reported.

A further increase in contact time did not result in further increase in the amount of Ni(II) adsorbed. To determine the order of the reaction, the experimental data were fit to a pseudo-first-order equation and a pseudo-second-order equation.

The pseudo-first-order equation can be expressed as shown in Equation (6):

(6) $${\ln(q}_{e}{-\;q}_{t}{)\;=\;\mathrm{lnq}}_{e}{-\;k}_{1}t$$

where *q_e_* and *q_t_* are the adsorbed amounts of metal ions at equilibrium and at time *t*, respectively (mg g^–1^), t is the contact time (min), and *k*_1_ is the pseudo-first-order adsorption rate constant (L min^–1^).

The plot of ln(*q_e_ − q_t_*) versus time (*t*) should be linear, and the rate constant *k_1_* was obtained from the slope. The calculated values obtained from the linear plots did not agree with the experimental *q*_e_ values, and the obtained *R*^2^ values were lower than the values of the pseudo-second-order kinetic model (Table 6), indicating that the adsorption of Ni(II) on bentonites JP and L did not follow the pseudo-first-order kinetic model.

The kinetics data were also fit to the pseudo-second-order equation (proposed by Ho and McKay [46]), expressed by Equation (7):

(7) $$\frac{t}{q_{t}}\;=\;\frac{1}{k_{2}\cdot {\;q}_{e}{}^{2}}\;+\;\frac{1}{q_{e}}t$$

where *k_2_* is the pseudo-second-order adsorption rate constant (g mg^–1^ min), calculated from the slope of the linear plot of *t*/*q*_t_ against *t.*

**Figure 7:**
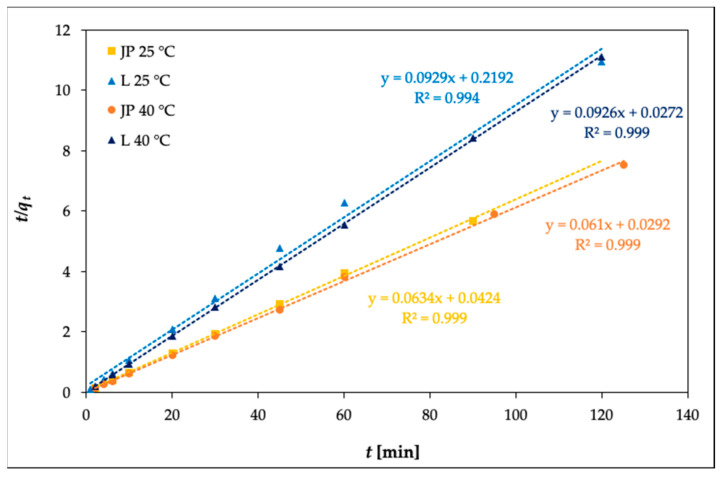
Pseudo-second-order adsorption kinetics of Ni(II) on bentonites JP and L (*c_0_* = 100 mg L^–1^).

Figure 7 illustrates the pseudo-second-order kinetics of Ni(II) adsorption onto bentonites JP and L at temperatures of 25 and 40 °C, respectively.

From the data given in Table 6, *R*^2^ for the pseudo-first-order model (*R^2^* = 0.1001–0.6706) are much smaller than those of the pseudo-second-order model (*R^2^* = 0.9993–0.9999) for Ni(II) adsorption. The *q_e_* values obtained from the pseudo-second-order model were in good agreement with the experimental data. This means that the adsorption of Ni(II) is based on the assumption that the rate-limiting step may be chemisorption and that adsorption closely follows the pseudo-second-order kinetic model [41].

### 3.5. Thermodynamic Studies

The effect of the temperature on the adsorption of Ni(II) onto natural bentonites was studied at 20, 30, and 40 °C. It was found that the increase in temperature had a favorable effect on the adsorption process, as with increasing temperature the amount of Ni(II) adsorbed on the bentonite also increased.

**Figure 8:**
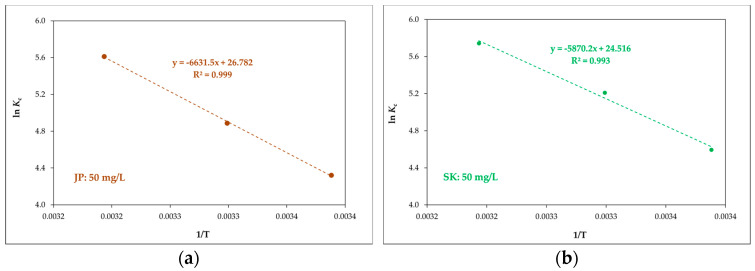
Linear plots of *K*c versus 1/T for JP (**a**) and SK (**b**) bentonite and for a Ni(II) concentration of 50 mg L^–1^.

The equilibrium constant *K_c_* and the thermodynamic parameters (Gibbs free energy Δ*G^0^* (kJ·mol^–1^), enthalpy Δ*H^0^* (kJ·mol^–1^), and entropy change Δ*S^0^* (J·mol^−1^·K^−1^))were calculated using Equations (8) and (9):

(8) $${\mathrm{lnK}}_{c}\;=\;\frac{{\Delta S}^{0}}{R}\;-\;\frac{{\Delta H}^{0}}{R\cdot T}$$

(9) $${\Delta G}^{0}{\;=\;-\mathrm{RTlnK}}_{c}$$

where *T* is the temperature (K), *R* is the ideal gas constant (8.314 J·mol^−1^·K^−1^), K_c_ is the ratio of the equilibrium concentration of the Ni(II) attached to bentonite to the equilibrium concentration in the solution and was determined from Langmuir adsorption isotherms, and the Langmuir constant *b* was calculated according to Milonjić [47]. The values of Δ*H^0^* and Δ*S^0^* were determined from the slopes and intercepts of the linear plots of ln *K_c_* versus 1/*T* (Figure 8) and are shown in Table 7. The values of Δ*G^0^* were calculated using Equation (9).

The enthalpy Δ*H^0^* is defined as a measure of the energy barrier that needs to be overcome by the reaction of molecules [48]. The Δ*H^0^* values (Table 7) are positive, indicating that a large amount of heat was consumed in transferring Ni(II) from the liquid to the solid phase. The positive values of Δ*H^0^* suggest that Ni(II) adsorption is endothermic, as reported elsewhere [27].

The changes in entropy (Δ*S^0^*) were 222.7, 203.8, 49.1, and 26.4 J·mol^−1^·K^−1^for the JP, SK, L, and S bentonites, respectively. The positive values of Δ*S^0^* show that the adsorption process is irreversible. The change in entropy Δ*S^0^* higher than −10^−2^ kJ·mol^−1^·K^−1^ indicated that the adsorption process takes place via a dissociation mechanism [16].

The degree of spontaneity and feasibility of an adsorption process is described by the Gibbs free energy (Δ*G^0^*). The values of Δ*G^0^* were negative, confirming that the adsorption of Ni(II) onto the bentonites used in this work is a spontaneous and feasible process. More negative Δ*G^0^* values imply a greater driving force for the adsorption process.

The values of Δ*G^0^* decreased with the increasing temperature (as shown in Table 4). This indicated that the reaction is more favorable and spontaneous at higher temperatures, similar to the behavior reported previously [27].

## 4. Conclusions

In this study, the removal of nickel via adsorption onto natural bentonites from four different Slovak deposits was investigated in detail. Batch adsorption experiments were conducted at various initial concentrations of Ni(II), times, and temperatures.

The adsorption isotherms were described by the Langmuir isotherm model and by the Freundlich isotherm model. The maximum adsorption capacity for the bentonites used decreased following the order of SK > JP > L > S. The data obtained from the kinetic study best fitted the second-order kinetic model. The thermodynamic data indicated that the adsorption process was endothermic and spontaneous.

Based on the results obtained from this study, it can be concluded that of all used bentonites, the bentonites from Central Slovakia (Jelšový Potok and Stará Kremnička) are most suitable for the removal of nickel from aqueous solutions.

## Figures and Tables

**Table 1:** Chemical analysis of bentonites [24,25,28,29,30,31,32].

Sample	SiO_2_ (%)	Al_2_O_3_ (%)	Fe_2_O_3_ (%)	TiO_2_ (%)	CaO (%)	MgO (%)	K_2_O (%)	Na_2_O (%)
JP	67.4	21.3	2.8	0.1	1.8	4.2	1.0	0.4
L	63.4	22.3	8.2	0.9	1.1	1.6	1.7	0.5
S	70.8	17.5	3.7	0.2	2.6	2.4	1.6	0.5
SK	56.5	18.2	2.1	0.2	1.6	2.5	1.4	0.3

**Table 2:** Mineralogical compositions of bentonites [24,25,28,29,30,31,32].

Sample	Smectite (%)	Quartz (%)	Biotite (%)	Kaolinite (%)	Opal-C (%)	Feldspars (%)
JP	86	6	1	2.5	* b.d.	3.5
L	64	9.5	2	9.5	b.d.	9.5
S	47	3	3	b.d.	20	12
SK	85	2	3	b.d.	5	5

* b.d.: below the detection limit.

**Table 3:** Parameters of Langmuir and Freundlich isotherms.

Adsorbent	Temperature (°C)	Langmuir Parameters			Freundlich Parameters		
		*q_m_* (mg g^–1^)	*b* (L mg^–1^)	*R^2^*	*K_f_*	1/*n*	*R^2^*
JP	20	21.79 ± 0.65	0.417 ± 0.013	0.999	10.170 ± 0.305	0.173 ± 0.005	0.999
	30	23.75 ± 0.71	0.264 ± 0.008	0.992	10.864 ± 0.326	0.161 ± 0.005	0.985
	40	25.45 ± 0.76	0.359 ± 0.011	0.994	12.128 ± 0.364	0.153 ± 0.005	0.959
L	20	12.24 ± 0.36	0.106 ± 0.003	0.997	4.376 ± 0.131	0.203 ± 0.006	0.983
	30	14.22 ± 0.43	0.056 ± 0.002	0.988	3.749 ± 0.112	0.250 ± 0.008	0.999
	40	13.00 ± 0.39	0.136 ± 0.004	0.997	5.175 ± 0.155	0.187 ± 0.006	0.979
S	20	8.41 ± 0.25	0.095 ± 0.003	0.999	3.581 ± 0.107	0.163 ± 0.005	0.922
	30	9.54 ± 0.29	0.052 ± 0.002	0.998	2.553 ± 0.077	0.242 ± 0.007	0.999
	40	8.58 ± 0.26	0.125 ± 0.004	0.999	4.281 ± 0.128	0.131 ± 0.004	0.996
SK	20	21.93 ± 0.66	0.516 ± 0.015	0.999	10.837 ± 0.325	0.163 ± 0.005	0.994
	30	25.00 ± 0.75	0.350 ± 0.011	0.995	11.668 ± 0.350	0.164 ± 0.005	0.992
	40	25.64 ± 0.77	0.592 ± 0.018	0.998	12.987 ± 0.389	0.158 ± 0.005	0.999

**Table 4:** *R_L_* values for bentonites at concentrations of 50–300 mg L^–1^ Ni(II).

Adsorbent	Temperature (°C)	Concentration (mg L^–1^)			
		50	100	200	300
JP	20	0.0561 ± 0.0017	0.0293 ± 0.0009	0.0152 ± 0.0005	0.0106 ± 0.0003
	30	0.0671 ± 0.0020	0.0344 ± 0.0010	0.0175 ± 0.0005	0.0119 ± 0.0004
	40	0.0039 ± 0.0001	0.0020 ± 0.0001	0.0010 ± 0.0001	0.0007 ± 0.0001
L	20	0.1194 ± 0.0036	0.0643 ± 0.0019	0.0341 ± 0.0017	0.0239 ± 0.0007
	30	0.1534 ± 0.0046	0.0822 ± 0.0025	0.0429 ± 0.0013	0.0295 ± 0.0009
	40	0.0033 ± 0.0001	0.0016 ± 0.0001	0.0009 ± 0.0001	0.0006 ± 0.0001
S	20	0.0927 ± 0.0028	0.0493 ± 0.0015	0.0259 ± 0.0008	0.0181 ± 0.0005
	30	0.4759 ± 0.0143	0.3100 ± 0.0093	0.1833 ± 0.0055	0.1323 ± 0.0040
	40	0.1404 ± 0.0042	0.0775 ± 0.0023	0.0410 ± 0.0012	0.0297 ± 0.0009
SK	20	0.0396 ± 0.0012	0.0205 ± 0.0006	0.0106 ± 0.0005	0.0074 ± 0.0004
	30	0.0532 ± 0.0016	0.0271 ± 0.0008	0.0137 ± 0.0007	0.0094 ± 0.0005
	40	0.0039 ± 0.0001	0.0020 ± 0.0001	0.0010 ± 0.0001	0.0007 ± 0.0001

**Table 5:** Comparison of the maximum adsorption capacities (*q*_max_) of different natural and modified bentonites for the Ni(II) removal.

Adsorbent	*q*_max_ (mg g^–1^)	Reference
Bentonite	9.91 (25 °C)	[40]
Bentonite	14.4 (22 °C)	[41]
Natural Ca-bentonite	26.32	[42]
Nano-bentonite	39.06 (30 °C)	[27]
Lieskovec bentonite	10.62	[25]
Lieskovec bentonite	12.24 (20 °C)	This study
Kopernica bentonite	22.01	[24]
Jelšový potok bentonite	18.43	[24]
Jelšový potok bentonite	21.79 (20 °C)	This study
Lastovce bentonite	14.09	[24]

**Table 6:** Comparison of the experimental and calculated *q_e_* values and the adsorption rate constants for the pseudo-first-order and pseudo-second-order reaction kinetics of the Ni(II) adsorption on the JP and L bentonites.

**Pseudo-first order**					
Samples	t(°C)	Experimental *q_e_* (mg g^–1^)	*k*_1_ (L min^–1^)	Calculated *q_e_* (mg g^–1^)	*R^2^*
JP	25	16.31	0.0151 ± 0.0008	1.83 ± 0.09	0.6706
	40	16.46	0.0072 ± 0.0004	0.42 ± 0.02	0.1001
L	25	9.81	0.0151 ± 0.0008	0.41 ± 0.02	0.1779
	40	10.81	0.0146 ± 0.0007	3.10 ± 0.16	0.3131
**Pseudo-second order**					
Samples	t(°C)	Experimental *q_e_* (mg g^–1^)	*k*_2_ (g mg^–1^ min)	Calculated *q_e_* (mg g^–1^)	*R^2^*
JP	25	16.31	0.0948 ± 0.0047	15.77 ± 0.79	0.9993
	40	16.46	0.1274 ± 0.0064	16.39 ± 0.82	0.9994
L	25	9.81	0.0394 ± 0.0019	10.76 ± 0.54	0.9998
	40	10.81	0.3153 ± 0.0158	10.80 ± 0.54	0.9999

**Table 7:** The values of thermodynamic parameters Δ*G^0^*, Δ*H^0^*, and Δ*S^0^*.

Adsorbent	t (°C)	Δ*G^0^* (kJ mol^–1^)	Δ*H^0^* (kJ mol^–1^)	Δ*S^0^* (J·mol^−1^·K^−1^)
JP	20	–10.72 ± 0.54	55.13 ± 1.65	222.67 ± 6.68
	30	–12.12 ± 0.61		
	40	–13.92 ± 0.70		
L	20	–2.77 ± 0.14	11.89 ± 0.36	49.05 ± 1.47
	30	–2.64 ± 0.13		
	40	–3.44 ± 0.17		
S	20	–1.00 ± 0.05	7.36 ± 0.22	26.42 ± 0.79
	30	–0.56 ± 0.03		
	40	–1.38 ± 0.07		
SK	20	–11.39 ± 0.57	48.80 ± 1.46	203.83 ± 6.11
	30	–12.92 ± 0.65		
	40	–14.24 ± 0.71		

## Data Availability

The authors confirm that the data supporting the findings of this study are available within the article and supplementary material.

## Supplementary Materials

The following are available online at https://www.mdpi.com/1996-1944/14/2/282/s1, Figure S1: IR spectra of used bentonites after adsorption of Ni(II) ions.
