# Supplementary material for: Removal of Nickel from Aqueous Solutions by Natural Bentonites from Slovakia
Source: Materials (Basel). 2021 Jan 7;14(2):282. doi: 10.3390/ma14020282 (PMC7827643; doi:10.3390/ma14020282)
Supplement: Supplementary file 1 [file materials-14-00282-s001.pdf]

materials

Supplementary materials

Removal of Nickel from Aqueous Solutions by Natural Bentonites from Slovakia

Matej Šuránek [1], Zuzana Melichová [1,*], Valéria Kureková [2], Ljiljana Kljajević [3] and Snežana Nenadović [3]

1 Department of Chemistry, Faculty of Natural Sciences, Matej Bel University, Tajovskeho 40, 97401 Banská Bystrica, Slovakia; matej.suranek@student.umb.sk
2 Department, Institute of Inorganic Chemistry, Slovak Academy of Sciences, Dubravska cesta 9, 84536 Bratislava, Slovakia; valeria.bizovska@savba.sk
3 Department of Materials, Vinča Institute of Nuclear Sciences-National Institute of the Republic of Serbia, University of Belgrade, Belgrade 11000, Serbia; ljiljana@vin.bg.ac.rs (L.K.); msneza@vin.bg.ac.rs (S.N.)
* Correspondence: zuzana.melichova@umb.sk; Tel.: +421-48-446-7350

Citation: Šuránek, M.; Melichová, Z.; Kureková, V.; Kljajević, L.; Nenadović, S. Removal of Nickel from Aqueous Solutions by Natural Bentonites from Slovakia. *Materials* **2021**, *14*, 282. https://doi.org/10.3390/ma14020282

Received: 8 December 2020
Accepted: 5 January 2021
Published: 7 January 2021

Publisher's Note: MDPI stays neutral with regard to jurisdictional claims in published maps and institutional affiliations.

Copyright: © 2021 by the authors. Submitted for possible open access publication under the terms and conditions of the Creative Commons Attribution (CC BY) license (http://creativecommons.org/licenses/by/4.0/).

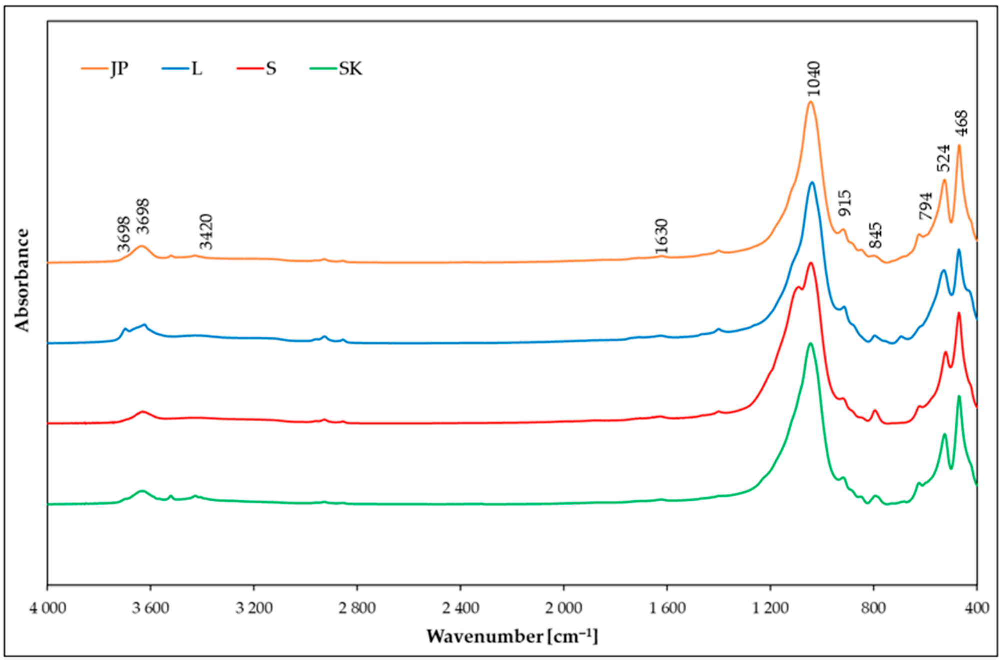

Figure S1. IR spectra of used bentonites after adsorption of Ni(II) ions.
